# Effectiveness of blood flow restriction versus traditional weight-bearing training in rehabilitation of knee osteoarthritis patients with MASLD: a multicenter randomized controlled trial

**DOI:** 10.3389/fendo.2023.1220758

**Published:** 2023-12-14

**Authors:** Chengfang Hu, Bin Zhu, Yanmao Wang, Fei Yang, Jun Zhang, Wanrun Zhong, Shengdi Lu, Congfeng Luo

**Affiliations:** ^1^ Department of Orthopedics, Shanghai Sixth People’s Hospital Affiliated to Shanghai Jiao Tong University School of Medicine, Shanghai, China; ^2^ Department of Orthopedics, The People’s Hospital of Mengla County, Mengla, China

**Keywords:** knee osteoarthritis, weight-bearing training, blood flow restriction, MASLD, rehabilitation

## Abstract

To compare the reliability and effectiveness of blood blow restriction resistance training (BFR) versus traditional weight-bearing training (WB) in knee osteoarthritis (KOA) patients with metabolic dysfunction-associated steatotic liver disease (MASLD).

**Methods:**

This multicenter randomized controlled trial was conducted from January 2021 to June 2022 at Shanghai Jiao Tong University affiliated Sixth People’s Hospital and The People’s Hospital of Mengla County. A total of 120 outpatients were recruited and randomized to perform WB (n=60) or BFR (n=60) resistance training protocols in accordance with standard recommended protocols for 12 weeks. Demographic data and Kellgren and Lawrence grading system scores were collected. Pain, range of motion (ROM), scaled maximal isotonic strength (10RM), self-reported function (KOOS), and 30-s chair sit-to-stand test results were assessed at weeks 1, 4, and 12.

**Results:**

112 patients (57 in the WB group, 55 in the BFR group) completed the training programs and assessments. No significant intergroup demographic differences were noted. ROM and scaled 10RM significantly increased at the 4- and 12-week assessments and differed significantly between groups. The pain, ability of daily living and quality of life subscale in KOOS increased significantly at the 12-week assessment and differed significantly between groups, adjusted for baseline value. Significant and comparable increases in 30-s chair sit-to-stand test results were observed within and between study groups.

**Conclusion:**

BFR training enhanced muscle strength, reduced pain, and improved daily living and sports activities in patients with KOA, compared to WB training alone. BFR should be recommended for rehabilitation in KOA individuals with MASLD.

**Clinical trial registration number:**

ChiCTR2100042872.

## 1 Introduction

Knee osteoarthritis (KOA), especially those cases involving the medial compartment of the tibiofemoral joint, has become a major public health problem worldwide (1, 2). As the main symptom of KOA pain continues to worsen as the disease progresses, eventually affecting knee joint function and ultimately affecting activities of daily living. The main treatment option for KOA is total knee arthroplasty (TKA), which incurs high cost (3). The obesity epidemic and its associated comorbidities including KOA and MASLD present a looming challenge to health care deliver (4). There is growing evidence that links these diseases to the systemic effects of adipose tissue dysfunction, which is partly due to excessive secretion of adipokines, fibrotic inductive factors, inflammatory cytokines, and lipids (5). In many patients, knee arthritis causes significant social and economic burdens (6). Therefore, we must establish effective conservative treatments to manage high-risk patients, including those with MASLD, to reduce KOA-induced burdens upon them effectively.

Exercise therapy can reduce the pain symptoms of KOA at different disease stages (7). Although studies have shown that the treatment duration approaches that of currently used drugs, the exact amount of exercise, frequency, and intensity still need to be determined. This may be because most randomized controlled trials published to date utilized general exercise therapy prescriptions but no individualized customizations of patients’ actual situations (8). Among patients with knee arthritis, strengthening the extensor knee muscle is crucial in reducing knee pain and functional limitations, and it is generally believed that resistance using a 12-maximum load (12RM) can promote myohypertrophy and enhance muscle strength (9, 10).

Blood flow restriction (BFR) training is a new strength training method that stimulates muscle growth and improves muscle function under proximal limb blood flow restriction or short-term intermittent blocking of venous blood flow during strength exercises performed with a small external load intensity (11). Joint pain may prevent some patients with KOA from completing high-resistance muscle strength training (12). Low- to moderate-resistance exercise is recommended for patients with KOA to reduce knee pain. However, this may limit increases in skeletal muscle strength (13, 14). BFR resistance exercises have recently been proposed to treat KOA (15, 16). Anti-BFR exercise is usually performed at a lower load via pneumatic cuff expansion to reduce arterial flow and limit venous return, thereby enhancing metabolic stimulation in the working muscle (17).

However, the effectiveness of BFR training (versus traditional WB training) for stimulating muscle strength in KOA patients with MASLD is unknown. Moreover, the effect of BFR on other aspects important in KOA rehabilitation, such as physical function and pain, has not been explored. Therefore, this study aimed to compare the reliability and effectiveness of BFR with traditional WB training in KOA patients with MASLD.

## 2 Methods

### 2.1 Participants

This multicenter randomized controlled trial was conducted from January 2021 to June 2022 in two trauma centers (Shanghai Jiao Tong University affiliated Sixth People’s Hospital and The People’s Hospital of Mengla County). A total of 120 eligible outpatients were recruited for this two-arm single-assessor blinded randomized control trial.

Inclusion criteria included:

a) Age ≥ 50 years.
b) Chief complaint of persistent or recurrent knee pain in the past few months.
c) A history of knee pain ≥ 3 months.
d) X-ray showing bony joint degeneration of the tibial joint.
e) Meet the diagnostic criteria of MASLD.
f) Ability and willingness to communicate daily using WeChat (Tencent Tech, Shenzhen, China).

Exclusion criteria included:

a) Record or plan of knee surgery/intra-articular injection within the past or next 6 months.
b) Steroid use within the previous 4 weeks.
c) Autoimmune arthritis.
d) History of knee fracture or lower-limb deformity.
e) History of knee/hip replacement or osteotomy.
f) Diseases associated with other imaged lower limb functions.
g) Inability to walk normally.
h) Inability to communicate normally or poor medical compliance.

MASLD was confirmed based on the diagnostic criteria (18): Hepatic steatosis identified by imaging and the presence of at least one of the five cardiometabolic risk factors and there are no other causes of hepatic steatosis: (1) BMI ≥ 23 kg/m^2^ or waist circumference > 94 cm in men or > 80 cm in women; (2) fasting serum glucose ≥ 5.6 mmol/L or 2h plasma glucose levels ≥ 7.8 mmol/L or glycated hemoglobin A1c ≥ 5.7% or type 2 diabetes or treatment for type 2 diabetes; (3) blood pressure ≥ 130/85 mmHg or antihypertensive drug treatment; (4) triglycerides ≥ 1.70 mmol/L or lipid lowering treatment; or (5) high-density lipoprotein cholesterol ≤ 1.0 mmol/L in men or ≤ 1.3 mmol/L in women or lipid lowering treatment.

All patients provided written informed consent in compliance with the declaration of Helsinki before joining the study. All study protocols were approved by the Research Ethics Committee of Shanghai Jiao Tong University affiliated Sixth People’s Hospital (REC reference no.: 2016-110). This clinical trial was registered at chichr.org.cn (ID: ChiCTR2100042872).

### 2.2 Sample size

The primary outcome measure of pain (Knee Injury and Osteoarthritis Outcome Score [KOOS] pain) was used to calculate the required sample size using G*power version 3.1 (Heinrich-Heine-Universität Düsseldorf Universitätsstr, Germany) (19). The minimal detectable change in KOOS pain was based on the values in a recent report (20). To achieve a power of 95% at an alpha level of 0.05, a total of 96 patients (48 per group) was required. Thus, to account for up to a 20% drop-out rate, a total of 120 patients were recruited.

### 2.3 Experimental design

Two trauma centers joined this two-arm, single-assessor blinded randomized controlled trial. All recruited patients were block randomized to the WB (n=60) or BFR (n=60) group by an independent research team member. The blinding procedure was performed using opaque envelopes (n=60 for each group). Each participant was asked by an independent research team member to choose one of the 120 envelopes. The groups were coded by an independent member of the research team, while the principal assessor of the results and data analysis was blinded to the group allocations.

### 2.4 Experimental procedure

**Figure 1:**
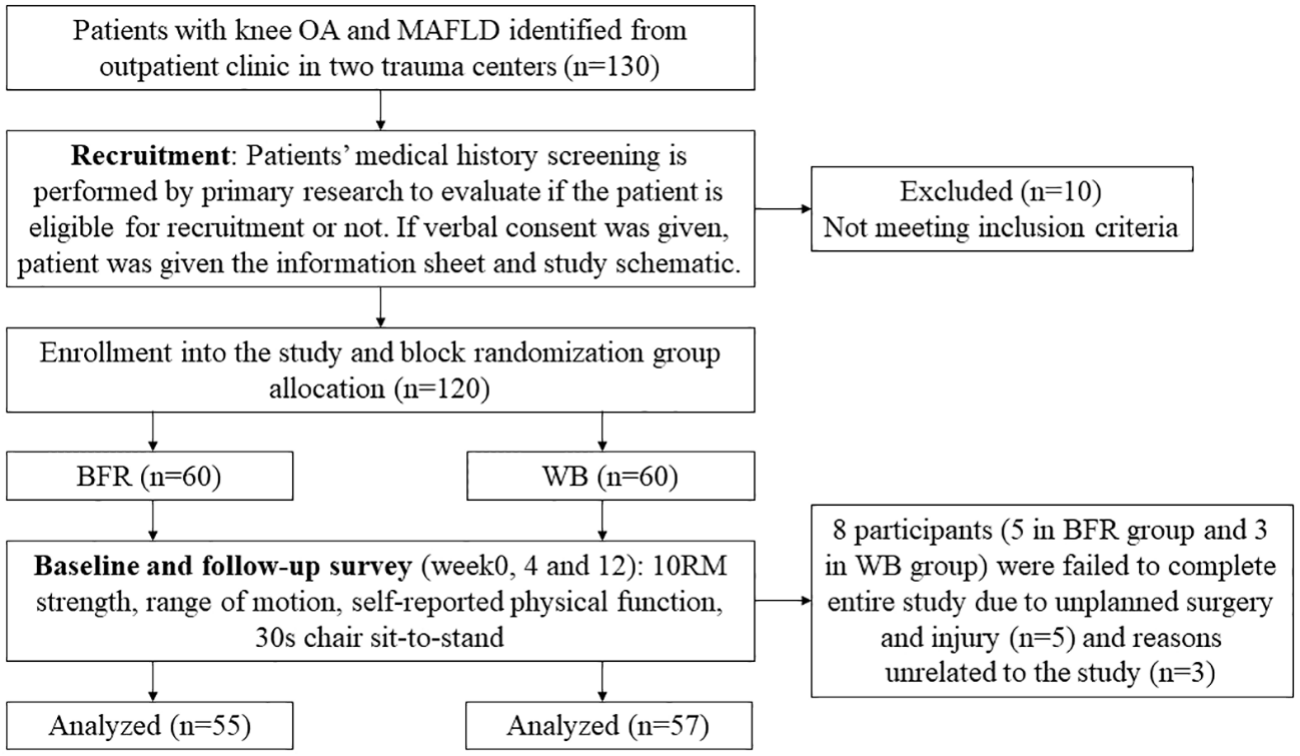
Trial Profile of WB vs BFR in participants.

The patients’ demographic data, including age, sex, BMI, smoking, diabetes, and Kellgren and Lawrence system grade, were recorded, as were their baseline data, including ROM, muscle strength (scaled maximal isotonic strength [10RM]), KOOS subscales, and 30-s chair sit-to-stand test results. All participants were then instructed to complete 12 weeks of training and undergo assessments at 4 and 12 weeks (**Figure 1**).

### 2.5 WB training

Each WB training intervention session included three parts: stretching exercise, ROM exercise, and strength exercise. The stretching exercises included triceps stretching in the standing position, hamstring stretching in the supine position, and quadriceps stretching in the prone position. The ROM exercises included knee extension at the end of the long sitting position, knee flexion at the end of the long sitting position, and stationary bike or trainer use. The strength exercise included quadriceps activation in the knee extension position and advanced closed-chain training of the lower limb. All training sessions were supervised by a physical therapist from the research team. Both groups were designed according to standard recommended protocols for each exercise type (**Table 1**).

**Table 1:** Rehabilitation training program.

Training content	Training movements	Requirements
Stretch exercise	Triceps calf stretching in standing position	Repeat 3 times, holding each time for 30 seconds
	Hamstring stretching in supine position	
	Quadriceps stretching in prone position	
Joint range of motion exercises	Knee extension at the end of the long sitting position	Continuous knee movement for 30 seconds, of which 3 seconds hold at the end, repeat 2 times
	Knee flexion at the end of the long sitting position	Continuous knee movement for 30 seconds, of which 3 seconds hold at the end, repeat 2 times
	Stationary bikes or trainers	For a maximum of 10 minutes, increase the ride time gradually according to tolerance
Strength training	Quadriceps muscle activation in knee extension position	Held for 6 seconds per contraction with 10 repetitions per group, repeat 3 times
	Advanced training of lower limb closed chain: A. hip bridge B. Squat with 1/4 leg C. Half squat with legs D. Lunge 1/4 squat E. lunge half squat F. Step-up function exercise G. Step-down function	Exercise A-G According to the patient’s condition, select 3 exercises that do not cause pain each time, and provide appropriate resistance to ensure that each group can carry out 12-15 repetitions without pain. If the patient is unable to complete 12-15 repetitions, the maximum number of repetitions is performed. Do 3 sets of exercises for each

### 2.6 BFR training

An automatically personalized tourniquet (PT) system (Delfi Medical, Vancouver, BC, Canada) was used in the BFR group. The system, set to automatically calculate occlusion pressure of the lower limb (LOP) defined as the minimum pressure required for full arterial occlusion (21), has clinically acceptable accuracy and high reliability (22–24). The PT device increases the cuff pressure in a step-up fashion, analyzes the pressure pulsation in the cuff bladder from the arterial pressure pulsation at each increase, and uses these properties to determine the LOP. The system consists of an easy-to-install dual-purpose variable profile nylon cuff (11.5 cm × 86 cm; 5 mm thick) that connects to the PT system via a sealing hose and automatically adjusts the pressure to within acceptable limits. Before the exercise, the cuff was placed at the proximal end of the limb and LOP was calculated in the position stimulated by the BFR. The BFR pressure was set at 80% LOP to maximize the recruitment of fast-twitch fibers and maximize muscle adaptation (25, 26). The LOP of each limb was computed separately for each session.

### 2.7 Pain

The pain was evaluated using the pain subscale of the KOOS (9-question, with a score of 0 indicating extreme pain to 36 indicating no pain).

### 2.8 Range of motion

Knee flexion was measured using a goniometer with each patient positioned supine and moving the heel as close as possible to the buttocks. Knee extension was measured with the patient maximally extending the knee joint and recorded as the difference from 0° of extension. The ROM of the knee was calculated as maximum flexion degree – maximum extension degree.

### 2.9 Muscle strength

Scaled 10RM strength was assessed using a MED leg press (Technogym, Bracknell, UK) following a warm-up of 10 min light cycling. Beginning at 80% estimated 10RM the maximum load that could be lifted ten times through complete, the full ROM performed using the correct form was recorded as the concentric 10RM. All 10RM values were achieved within five attempts at 5-kg increments in external load at each attempt and a 3-min rest between attempts to allow full muscle recovery. The 10RM load for each patient was established at a level where they were capable of completing the 10th repetition but unable to perform an 11th repetition. Leg press exercise techniques followed the recommendation of the National Strength and Conditioning Association (27).

### 2.10 Physical function

The KOOS is a self-reported tool used to assess patients’ opinions of their knee function and associated problems. The KOOS has five subscales: pain, symptoms, function in daily living, function in sport and recreation, and knee-related quality of life. Each subscale includes questions with standardized answer options across five Likert boxes scored 0–4. Each subscale was scored independently, with 0 indicating maximum symptoms.

The 30-s chair sit-to-stand test (30s-CST) test is the American College of Sports Medicine (ACSM) recommended function and strength measurement test for elderly individuals. Participants were asked to stand from the chair and sit down as many times as possible in 30 s. The MDC value of the 30s-CST is 1.13 s (28).

### 2.11 Data storage and analysis

All patients’ data were coded and stored on the electronic data capture system for a specific disease. The system was operated within the hospital’s local servers. All statistical analyses were performed using SPSS Statistics version 24.0 (IBM Corp, Chicago, IL, USA). Data are presented as mean ± SD with 95% confidence intervals unless stated otherwise. Intergroup differences in patients’ baseline characteristics were assessed using independent-sample t-tests for continuous dependent variables and Fisher’s exact test for categorical data. The Shapiro-Wilks test (p>0.05) was used to examine normally distributed data, while Levene’s test of homogeneity was used to examine the homogeneity of the variances. The analysis of variance test was used to assess pain, 10RM strength, self-reported function, and 30s-CST with group allocations as the inter-subject independent factor and time as the intra-subject dependent factor.

## 3 Results

### 3.1 Participants and rehabilitation program

Eight participants (5 in the BFR group, 3 in the WB group) failed to complete the study due to unplanned surgery and injury (n=5) and unrelated reasons (n=3). The remaining 112 participants (93%) completed the study and the follow-up survey. There were no significant intergroup differences in baseline demographic data (**Table 2**), including adherence or protocol changes. No adverse events were reported.

**Table 2:** Baseline characteristics of the participants.

	BFR (n=55)	WB (n=57)	*P*-value
Age(years)	67.2 ± 8.2	67.1 ± 7.7	0.947
Gender (male/female)	24/31	27/30	0.692
BMI (kg/m^2^)	28.6 ± 2.7	27.9 ± 2.8	0.181
Diabetes (%)	30.9% (n=17)	33.3% (n=19)	0.784
Smoking (%)			0.419
Nonsmoker	60% (n=33)	56.1% (n=32)	
Former smoker	20% (n=11)	29.8% (n=17)	
Smoker	20% (n=11)	14% (n=8)	
KL grade (%)			0.252
Grade 2	41.8% (n=23)	52.6% (n=30)	
Grade 3	58.2% (n=32)	47.4% (n=27)	

Continuous data were presented as mean ± SD. Other data were reported as percentage (n).

### 3.2 Pain

Statistically significant improvement in KOOS pain scores were detected at the 4- and 12-week follow-up assessments, with significant intergroup differences at 12 weeks (**Table 3**). The change in KOOS pain score differ between groups from baseline to 4- and 12- weeks (**Table 4**).

**Table 3:** Results of pain, ROM, and physical function at baseline, week 4 and 12.

	BFR (n=55)	WB (n=57)	*P*-value
ROM (°)			
Baseline	107.3 ± 3.4	107.8 ± 4.9	0.566
Week 4	127 ± 4.6	121.1 ± 3.7	< 0.01
Week 12	132.2 ± 4.2	127.1 ± 3.6	< 0.01
Scaled 10RM (kg/kg)			
Baseline	0.91 ± 0.24	0.93 ± 0.22	0.702
Week 4	1.19 ± 0.17	1.12 ± 0.14	0.02
Week 12	1.34 ± 0.16	1.21 ± 0.09	< 0.01
KOOS Pain			
Baseline	20.3 ± 4.3	20.3 ± 4.1	0.698
Week 4	22.6 ± 3.1	22.1 ± 3.4	0.358
Week 12	24.4 ± 2.5	22.9 ± 2.9	< 0.01
KOOS Symptoms			
Baseline	10.7 ± 3.4	10.2 ± 2.5	0.480
Week 4	14.1 ± 3.1	12.3 ± 2.1	< 0.01
Week 12	16.4 ± 2.3	14.9 ± 3.1	< 0.01
KOOS Ability of daily living			
Baseline	25.8 ± 3.9	25.7 ± 3.4	0.871
Week 4	30.9 ± 3.0	29.6 ± 2.9	0.019
Week 12	35.7 ± 2.4	32.4 ± 3.6	< 0.01
KOOS Sports and recreation			
Baseline	8.6 ± 1.9	7.8 ± 3.5	0.189
Week 4	9.8 ± 1.9	9.1 ± 3.1	0.024
Week 12	11.5 ± 1.5	10.1 ± 2.3	< 0.01
KOOS Knee-related quality of life			
Baseline	4.8 ± 1.9	4.9 ± 2.3	0.105
Week 4	5.9 ± 1.6	6.1 ± 1.8	0.621
Week 12	8.5 ± 2.3	6.9 ± 1.6	< 0.01
30s-CST (times)			
Baseline	11.2 ± 1.9	11.3 ± 2.1	0.837
Week 4	13.9 ± 1.8	13.1 ± 1.8	0.01
Week 12	17.2 ± 1.1	15.1 ± 1.4	< 0.01

Data were presented as mean ± SD. Kg/kg as a unit presented the ratio of the weight lifted to the body weight.

**Table 4:** Change of pain, ROM, and physical function in baseline, week 4 and 12.

	BFR (n=55)	WB (n=57)	*P*-value
ROM (°)			
From week 0 to 4	19.6 ± 5.2	13.2 ± 5.6	< 0.01
From week 4 to 12	5.2 ± 3.5	6.0 ± 3.5	0.210
From week 0 to 12	24.8 ± 4.6	19.2 ± 6.1	< 0.01
Scaled 10RM (kg/kg)			
From week 0 to 4	0.28 ± 0.16	0.19 ± 0.15	< 0.01
From week 4 to 12	0.15 ± 0.12	0.09 ± 0.11	< 0.01
From week 0 to 12	0.43 ± 0.18	0.28 ± 0.19	< 0.01
KOOS Pain			
From week 0 to 4	2.3 ± 1.4	1.8 ± 2.3	0.023
From week 4 to 12	1.7 ± 2.0	0.9 ± 4.3	0.182
From week 0 to 12	4.1 ± 3.1	2.6 ± 4.9	< 0.01
KOOS Symptoms			
From week 0 to 4	3.4 ± 2.6	2.1 ± 1.8	< 0.01
From week 4 to 12	2.2 ± 1.5	2.5 ± 3.1	0.486
From week 0 to 12	5.6 ± 2.7	4.6 ± 3.6	0.094
KOOS Ability of daily living			
From week 0 to 4	5.1 ± 3.2	3.8 ± 2.4	0.026
From week 4 to 12	4.7 ± 3.3	2.8 ± 3.5	< 0.01
From week 0 to 12	9.8 ± 4.8	6.7 ± 4.7	< 0.01
KOOS Sports and recreation			
From week 0 to 4	1.6 ± 1.6	1.2 ± 2.6	0.321
From week 4 to 12	1.3 ± 1.1	1.0 ± 2.7	0.469
From week 0 to 12	2.9 ± 1.7	2.2 ± 3.8	0.214
KOOS Knee-related quality of life			
From week 0 to 4	1.1 ± 1.4	1.2 ± 1.3	0.039
From week 4 to 12	2.6 ± 2.8	0.7 ± 1.0	< 0.01
From week 0 to 12	3.6 ± 3.2	1.9 ± 1.3	< 0.01
30s-CST (times)			
From week 0 to 4	2.6 ± 1.4	1.7 ± 1.0	< 0.01
From week 4 to 12	3.3 ± 2.0	2.0 ± 1.2	< 0.01
From week 0 to 12	6.0 ± 2.0	3.7 ± 1.7	< 0.01

Data were presented as mean ± SD. Kg/kg as a unit presented the ratio of the weight lifted to the body weight.

### 3.3 Range of motion

A statistically significant increase in knee ROM was noted at 4 and 12 weeks, with a significant intergroup difference (**Table 3**). The intergroup change in knee ROM from 0 to 4 and 0 to 12 weeks was significant (**Table 4**).

### 3.4 Scaled 10RM muscle strength

Scaled 10RM strength increased significantly from baseline to 12-week follow-up with significant intergroup differences (**Tables 3**, **4**).

### 3.5 Physical function

Statistically significant difference was noted for all KOOS subscale scores. The scores of subscales of symptoms, ability in daily living, function in sports and recreation, and knee-related quality of life showed significant intergroup differences at the 12-week follow-up (**Table 3**). The change of scores in subscales of daily living and knee-related quality of life differed significantly between groups at 0-4 weeks, 4-12 weeks, and 0-12 weeks (**Table 4**).

The mean 30s-CST increased significantly from baseline to 12-week follow-up with significant intergroup differences (**Tables 3**, **4**).

## 4 Discussion

This study was the first to assess the effects of a 12-week BFR training program on pain, muscle strength, and physical function among individuals with KOA complicated with MASLD. This study targeted middle-aged and elderly obese patients with a higher incidence of knee OA than other populations and aimed to evaluate the effectiveness of BFR in knee OA rehabilitation. The main findings of this study include: 1) WB training with or without BFR increases knee ROM and muscle strength in patients with KOA comorbid with MASLD; 2) pain reduction afforded by WB with BFR was greater than that of WB training in the short- (4 weeks) but not long-term (12 weeks); 3) WB with BFR improved function in daily living and knee-related quality of life better than WB training alone; 4) and WB and BFR are safe and reliable interventions for individuals with KOA comorbid with MASLD.

### 4.1 Pain

Different studies have evaluated the effects of training programs on pain, symptoms, physical function, and quality of life among patients with OA (29–31). Almost all evidence emphasized improvements in various aspects of patients’ daily lives following a regular exercise program. BFR is a novel auxiliary training method that reportedly improves the postoperative rehabilitation of anterior cruciate ligament reconstruction patients (32).

The subjects of this study were obese individuals with KOA who experience greater joint stress involving a cycle of pain and loss of strength and knee function due to damaged cartilage and altered joint mechanics. Heavy load training can be risky for such patients due to further cartilage degeneration. Brky et al. reported that patients experienced significantly less joint pain during BFR than heavy load training (33). The results of our study demonstrated the superiority of BFR over WB training alone in terms of long-term pain reduction (12 weeks) with no adverse events.

### 4.2 Muscle strength

Muscle strength and volume increases have been reported after all exercise protocols regardless of duration (4–12 weeks). Recent studies reported that 8–13% of knee extensor muscle strength could be recovered with the aid of BFR (34) (35), primarily due to the neuromuscular adaptations induced by the restricted blood flow. The hypoxic environment created by BFR induces greater recruitment of type II fibers and interleukin-6 and growth hormone concentrations (36, 37). Our results proved that BFR exercise for 12 weeks significantly increased muscle strength.

Arthrogenic inhibition is associated with joint cartilage impairments, effusion, and pain (38); therefore, the reduced KOOS pain scores observed in our study may have contributed to strength adaptations of the OA limb.

### 4.3 Physical function

The significant and clinically important improvements in all patient’s self-reported function measures and 30s-CST performance results observed in both groups are in line with those of recent studies of knee OA patients (14, 33). The improvements in physical function are attributable to improved strength and reduced pain. According to the KOOS subscale results, the pain, symptom, function in daily living, function in sports and recreation, and knee-related quality of life scores decreased significantly in both groups and differed significantly between them at the 12-week follow-up survey. Thus, the greater pain reduction and strength improvement may have contributed to improved ROM. A study examining a 3-week exercise program after TKA reported increased knee ROM and decreased knee pain (39). Thus, muscle strength training of the knee is suitable for the postoperative exercise of young patients undergoing anterior cruciate ligament reconstruction and the rehabilitation of obese patients with KOA. The American Academy of Orthopedic Surgeons, Osteoarthritis Research Society International, and American College of Rheumatology explain that strength increment promotes pain reduction, attenuates symptomatology, and reduces joint damage progression in individuals with KOA (40–42).

Recent research also suggested that BFR may have a hypoalgesia effect, as knee pain was significantly reduced during, immediately after, and 24 h after BFR versus heavy load resistance training (43, 44). Although the mechanism of this effect is unclear, there are several possibilities. Muscle pain due to ischemia and stress is often used as conditioned stimuli to modulate pain and can alter pain sensitivity in healthy individuals. Thus, structured pain modulation by BFR cuff pressure and exercise-induced muscle pain due to high ischemia levels and BFR resistance training may contribute to the antivaccination response. Other possible mechanisms include the release of endogenous opioids and endocannabinoids during exercise (45–47).

### 4.4 Implications for clinical knee OA rehabilitation

The application of BFR passively or in combination with aerobic exercise during early anterior cruciate ligament rehabilitation was discussed previously (13). The present study showed that BFR is also suitable for the rehabilitation of obese patients with KOA comorbid with MASLD, with the advantages that it affords a greater pain reduction and better improves physical function than WB exercise alone.

Pain extent is a major factor affecting knee OA rehabilitation results, as the pain has a detrimental effect on motor control and muscle function that results in modified movement patterns (48). The effects of pain reduction by BFR can improve the benefits of strength training. Therefore, BFR may be a superior tool during early KOA rehabilitation, particularly among patients reporting a high degree of pain.

### 4.5 Study strengths and limitations

During knee OA rehabilitation, applying progressively heavier loads is important to preventing muscular adaptations to the exercise (9). Our study did not use progressively heavier loads during the exercise protocol, which could have reduced the exercise’s effects. Further studies are needed to reveal the relationship between load progression and functional results.

Muscle morphology is another parameter used to evaluate functional outcomes of training. Still, it was not used in our study due to the 4.2–13.0 MHz wide-band linear array scanning transducer head and the image analysis software available to our study group.

BFR resistance exercise is employed by individuals with limited or incapacity of performing high-intensity resistance exercise, such as elderly (49) and orthopedic rehabilitation (13). Several studies offered caution to BFR resistance exercise (50–52), particularly regarding its cardiovascular effects. It’s suggested that individuals with conditions like heart failure, hypertension, or peripheral artery disease may lead to exaggerated metaboreflex activation during exercise (50), making BFR training potentially risky for them. There were no adverse events noted during the trial and follow-up. But still, careful prescription and monitoring are essential for safe practice, particularly in clinical populations.

This study included a specific subgroup of KOA patients, which limits the generalization of our findings to other patient populations. Moreover, it focused on a specific degree of KOA (Kellgren and Lawrence grade II–III), which may also limit the generalizability of our results.

## 5 Conclusion

The present study demonstrated that BFR training could improve knee strength better than WB training alone, affording a greater reduction in pain and leading to greater overall improvements in functional outcomes of daily living and sport and leisure for KOA patients with MASLD.

## Data availability statement

The raw data supporting the conclusions of this article will be made available by the authors, without undue reservation. Requests to access these datasets should be directed to luocongfeng@sjtu.edu.cn.

## Ethics statement

The studies involving humans were approved by the Research Ethics Committee of Shanghai Jiao Tong University affiliated Sixth People’s Hospital. The studies were conducted in accordance with the local legislation and institutional requirements. The participants provided their written informed consent to participate in this study.

## Author contributions

Conception and design: CH, CL. Acquisition, analysis, or interpretation of data: BZ, YW, FY, JZ, WZ, SL. Drafting: CH, SL. Revising: CL. All authors contributed to the article and approved the submitted version.
